# Surface Functional Poly(lactic Acid) Electrospun Nanofibers for Biosensor Applications

**DOI:** 10.3390/ma9010047

**Published:** 2016-01-14

**Authors:** Edurne González, Larissa M. Shepherd, Laura Saunders, Margaret W. Frey

**Affiliations:** 1 Department of Fiber Science and Apparel Design, Cornell University, Ithaca, NY 14853, USA; eg452@cornell.edu (E.G.); lb468@cornell.edu (L.M.S.); 2 Chemical and Biological Engineering Department, University at Buffalo, Buffalo, NY 14261, USA; laurasau@buffalo.edu

**Keywords:** biotin, avidin, poly(lactic acid) PLA, poly(lactic acid)-block-poly(ethylene glycol) (PLA-*b*-PEG), electrospinning, functional nanofibers

## Abstract

In this work, biotin surface functionalized hydrophilic non-water-soluble biocompatible poly(lactic acid) (PLA) nanofibers are created for their potential use as biosensors. Varying concentrations of biotin (up to 18 weight total percent (wt %)) were incorporated into PLA fibers together with poly(lactic acid)-block-poly(ethylene glycol) (PLA-*b*-PEG) block polymers. While biotin provided surface functionalization, PLA-*b*-PEG provided hydrophilicity to the final fibers. Morphology and surface-available biotin of the final fibers were studied by Field Emission Scanning Electron Microscopy (FESEM) and competitive colorimetric assays. The incorporation of PLA-*b*-PEG block copolymers not only decreased fiber diameters but also dramatically increased the amount of biotin available at the fiber surface able to bind avidin. Finally, fiber water stability tests revealed that both biotin and PLA-*b*-PEG, migrated to the aqueous phase after relatively extended periods of water exposure. The functional hydrophilic nanofiber created in this work shows a potential application as a biosensor for point-of-care diagnostics.

## 1. Introduction

Electrospinning is a well-established technique widely used to create continuous fibers with diameters ranging from a few micrometers down to tens of nanometers. This technique has gained extraordinary relevance in the last few decades due to its inexpensive nature and simplicity [1,2,3,4]. Electrospun polymeric nanofibers present unique properties, such as high surface area to volume ratio, porous structure and excellent pore interconnectivity, which make them attractive for a wide range of applications [1,4,5] including biosensors [6,7,8,9]. A small volume of electrospun fiber mat may provide a large surface area for sensing together with an easy access for the analyte to detection sites.

When nanofibers are used to construct biosensors, different important factors must be considered and combined; (1) surface functionality; (2) hydrophilicity; and (3) water solubility of the fibers. The nanofiber surface must be functional to interact with (or immobilize) biological molecules. Moreover, as most biological systems are aqueous, hydrophilic surfaces have shown to provide an increase in the materials function as biosensor as well as their biocompatibility [10,11]. Lastly, the fiber mat must be water insoluble to avoid its degradation in the aqueous phase.

Biotin and avidin (or streptavidin) binding has been broadly used to immobilize biologically active materials on surfaces in tissue engineering and biosensor applications [12,13,14,15]. Our research group has successfully synthesized biocompatible functional nanofibers incorporating biotin to poly(lactic acid) (PLA) nanofibers up to 18 weight total percent (wt %). These nanofiber membranes were able to immobilize streptavidin, which at the same time, was able to immobilize biotinylated nucleic acid probes and antibodies [6,16,17]. Li *et al*. [17] quantified the amount of incorporated biotin that was available for streptavidin binding at the PLA fibers surface by competitive colorimetric assays. They observed a non-linear increase of the surface available biotin compared to the overall biotin concentration, obtaining a maximum of 11% of the total incorporated biotin available at the fiber surface.

One of the major drawbacks of using PLA as a biosensor substrate is its high hydrophobicity, which has a negative impact in the wettability of the final nanofibers. A feasible method to increase the hydrophilicity of PLA is by blending it with a hydrophilic polymer [18,19,20,21,22]. Researchers have been able to increase the wettability of PLA nanofibers by the incorporation of poly(lactic acid)-*block*-poly(ethylene glycol) (PLA-*b*-PEG) block copolymers and PEG oligomers into the spinning dope [20,21]. During the electrospinning process, block copolymers and oligomers phase separated to create a fiber with hydrophilic PEG at the surface. Incorporation of PLA-*b*-PEG block copolymers was more efficient than PEG oligomers, resulting in fibers with higher homogeneity and wettability [20,21]. Buttaro *et al*. [21] studied the influence of block copolymer length and concentration, finding that the maximum wettability was obtained when PLA(1000)-*b*-PEG(5000) block copolymers were used at 12 wt % of PEG in the final fiber.

In this work, biotin surface functionalized hydrophilic PLA nanofibers were developed by the addition of different concentrations of biotin (up to 18 wt %) together with PLA-*b*-PEG block polymers to the spinning dope. For this purpose a modified electrospinning apparatus with a heating component [21] was used and the morphology, surface-available biotin and water stability of the final fibers was studied. The incorporation of the PLA-*b*-PEG block polymers to the fibers not only increased their wettability but also aided the migration of biotin to the surface producing a 506% increase of surface-available biotin.

## 2. Results and Discussion

### 2.1. Fiber Morphology by Field Emission Scanning Electron Microscopy (FESEM)

Two sample sets were prepared and their fiber morphology was analyzed by FESEM. In the first set, varying concentrations of biotin (0, 5, 10, and 18 wt % in the final fiber) were incorporated into PLA fibers by dissolving it in the initial PLA/dimethylformamide (DMF) spinning dope at elevated temperatures. DMF was chosen as solvent since it has shown to improve the dispersion of biotin in the final fiber [16]. In the second set, the same biotin concentrations were incorporated to the PLA/DMF spinning solutions with the addition of PLA-*b*-PEG block copolymers. The amount of PLA-*b*-PEG was adjusted to obtain 12 wt % of PEG in the final fibers in order to achieve maximum wettability [21].

**Figure 1:**
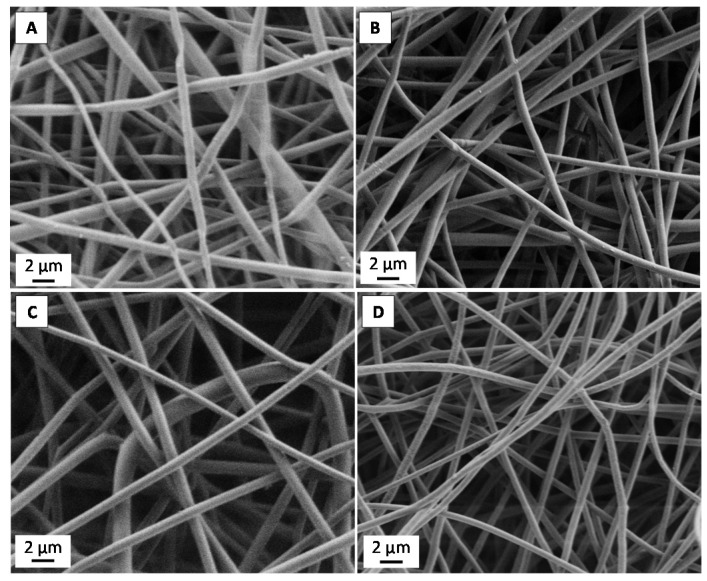
SEM images of PLA samples containing different amounts of biotin: (**A**) 0 wt %; (**B**) 5 wt %; (**C**) 10 wt % and (**D**) 18 wt %.

**Figure 2:**
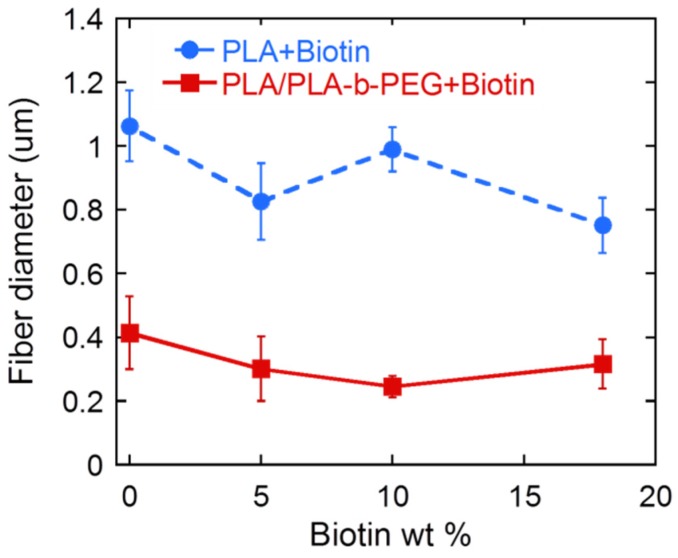
Average fiber diameter of PLA and PLA/PLA-*b*-PEG samples containing different amounts of biotin.

**Figure 3:**
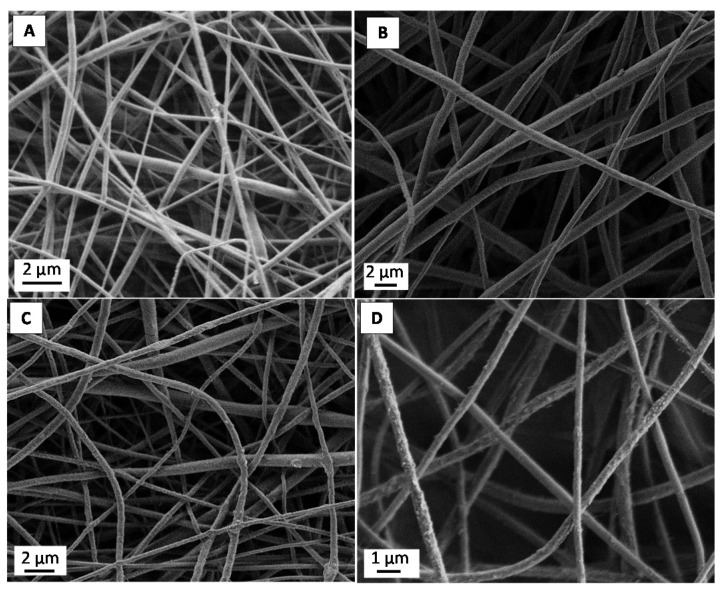
SEM images of PLA/PLA-*b*-PEG samples containing different amounts of biotin: (**A**) 0 wt %; (**B**) 5 wt %; (**C**) 10 wt % and (**D**) 18 wt %.

Fibers containing just biotin and PLA (Figure 1) show a smooth, bead-free and uniform morphology. No clear trend was observed in the mean average fiber diameters as the amount of biotin added increased (Figure 2). Thus, as previously observed by Frey and co-workers [16], addition of biotin to the spinning dope did not affect the final fiber morphology. When PLA-*b*-PEG copolymers were added (Figure 3), fibers appeared significantly smaller and again, no clear difference or trend was observed in the fiber diameter with the addition of biotin (Figure 2). Other authors also observed a decrease in fiber diameter when PLA-*b*-PEG or PEG oligomers were added to the spinning dopes [20,21,23,24,25] and was attributed to a decrease in the electrospinning solution viscosity.

### 2.2. Biotin Distribution and Measurement of Surface-Available Biotin

**Figure 4:**
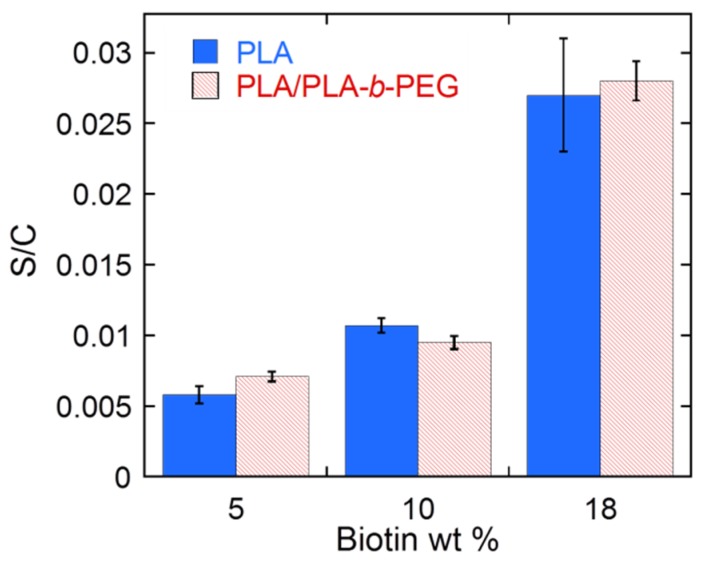
Sulfur/carbon (S/C) atom ratio of PLA and PLA/PLA-*b*-PEG samples with different biotin loading quantify by EDS analysis.

Energy dispersive spectroscopy (EDS) was used to analyze the distribution of biotin in the nanofiber mats. Five spectra were collected in random areas of each sample and the sulfur/carbon atom (S/C) ratio was quantified. Results are shown in Figure 4. As expected, the S/C atom ratio increased as the amount of biotin in the sample was increased. No statistically significant differences were observed between the results of PLA and PLA/PLA-*b*-PEG samples when 5 and 18 wt % of biotin was added and, more importantly, low standard deviation values were obtained in all cases, indicating that the amount of biotin was similar in all analyzed areas. Thus, it can be concluded that in both cases, PLA and PLA/PLA-*b*-PEG samples, biotin was well dispersed and the distribution of biotin in the fiber mats was mainly homogeneous.

**Figure 5:**
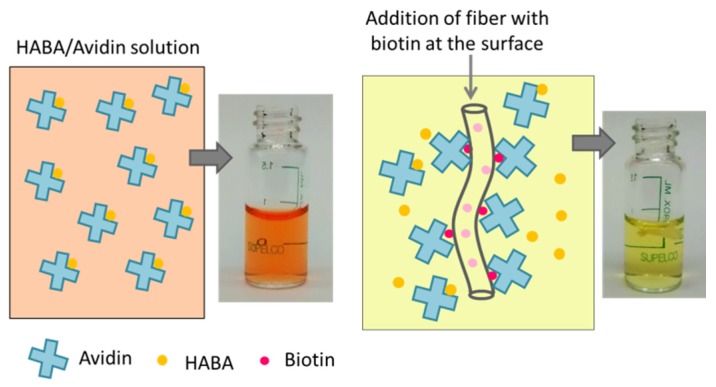
Illustrations and real pictures of the HABA/avidin solutions. Initially, HABA and avidin form a complex with a strong orange color (absorbs light at 500 nm). When a fiber containing biotin is added to the solution, avidin binds biotin due its higher affinity breaking the HABA/avidin complex and leading to a color change (decrease in the absorbance at 500 nm).

The penetration depth of the EDS analysis is on the micron scale (around 1 to 3 μm) and sulfur atoms associated with biotin in this analysis were located throughout the fiber thickness. As only the molecules located at the fiber surface are of interest, competitive colorimetric assays were used to quantify the amount of biotin available at the surface of the nanofibers [17]. The method consisted of adding a small, pre-weighed piece of fiber mat into a 4′-hydroxyazobenzene-2-carboxylic acid (HABA)/avidin solution and measuring the difference in the absorbance of the solution at 500 nm. HABA and avidin bind strongly to produce an orange colored complex that absorbs light at 500 nm. When a fiber mat containing biotin is added to the HABA/avidin complex solution, avidin is removed from the complex and binds with biotin due to its higher affinity, causing a decrease in the absorbance. Figure 5 shows the distinct visual color change between the orange HABA/avidin complex solution and the yellow HABA solution after avidin selectively binds to the biotin in the submerged fiber mat. It should be mentioned that no change in the absorbance of the HABA/avidin complex solution was observed when the control PLA and PLA/PLA-*b*-PEG samples (with 0 wt % biotin) were added. This indicates that the change in the absorbance of the solution can only be attributed to the presence of biotin at the fiber surface.

**Figure 6:**
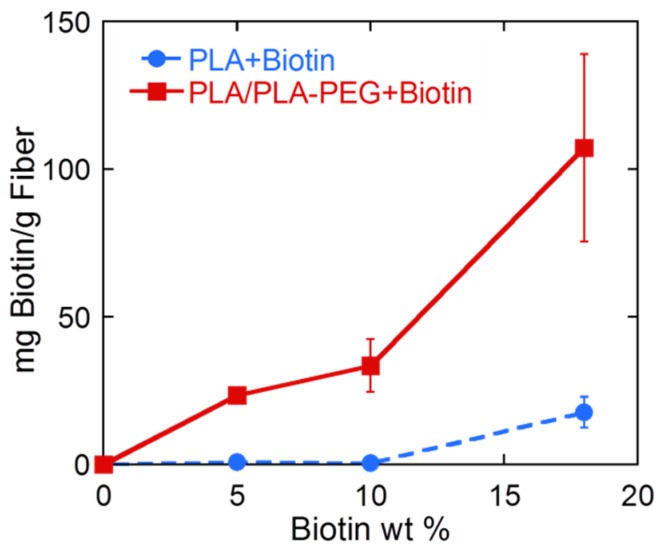
Surface available biotin of PLA and PLA/PLA-*b*-PEG samples containing different amounts of overall biotin.

Results of the colorimetric assays are shown in Figure 6. In the case of the PLA fibers, results correlate well with those reported by Li *et al*. [17]. Still in agreement with the previous work, the increment in the surface-available biotin was not linear regarding the total concentration of biotin added to the spinning dope. A small amount of surface-available biotin was measured in fibers containing an overall concentration of 5 and 10 wt % of biotin, 0.75 ± 0.18 and 0.50 ± 0.05 mg biotin/g fiber respectively. In these cases, only about 0.5%–1.5% of the overall amount of biotin added was present at the fiber surface. The amount of surface-available biotin drastically increased to 17.7 ± 5.2 mg biotin/g fiber when 18 wt % of biotin was added to the spinning dope, corresponding to 9.8% of the overall amount of biotin.

In the case of PLA/PLA-*b*-PEG fibers, the surface-available biotin was significantly higher than in PLA fibers for all biotin concentrations (Figure 6). Again, a non-proportional relationship was observed between the overall biotin concentration and the surface-available biotin. 23.4 ± 2.1 and 33.5 ± 8.9 mg Biotin/g fiber were measured in fibers containing 5 and 10 wt % of biotin, respectively, and a drastic increase to 107.2 ± 31.8 mg biotin/g fiber was observed when the overall amount of biotin was 18 wt %. Thus, when PLA-*b*-PEG is present, the addition of just 5 wt % of biotin leads to fibers with slightly higher surface-available biotin than when 18 wt % of biotin is added to PLA fibers. For PLA/PLA-*b*-PEG fibers containing 5, 10 and 18 wt % of biotin, 46.8%, 33.5% and 59.6% of the total biotin added was available at the surface respectively.

According to Li *et al.* [17], phase separation of biotin towards the fiber surface was driven by two main phenomena; the first one was the enhancement of the migration of polarizable species (biotin in this case) to the fiber surface due to electric field applied during the electrospinning process [26,27,28]. The second one was the lack of entropy of mixing in the polymer system that favors the phase separation of the components [29].

One of the parameters that could have influenced the colorimetric assay is the wettability of the sample. Penetration of HABA/avidin complex solution into the fiber mat might be easier in hydrophilic samples. Since the wettability of the PLA/PLA-*b*-PEG fibers was higher than the wettability of PLA fibers [21], several of the colorimetric assays for PLA samples were repeated increasing the wetting by the use of Tween 20 surfactant (Sigma-Aldrich, Milwaukee, WI, USA) [17]. Nevertheless, no significant differences were observed in the obtained results, proving that the low surface-available biotin measured in the case of PLA samples was not an artifact of the low wettability of the samples.

Another important parameter to be considered is the surface area of the sample. Therefore, in order to check if the increase in the surface-available biotin of the PLA/PLA-*b*-PEG samples was due to the enhanced migration of the biotin to the fiber surface or simply due to the larger surface area of these samples (as fiber diameter was smaller, the surface area of the samples was larger for the same sample mass), the amount of biotin molecules per square nanometer in the fibers surface was calculated (calculations are explained in the Supplementary Materials). Results are shown in Table 1. It can be clearly observed that PLA/PLA-*b*-PEG fibers contained more biotin molecules at their surface compared to PLA fibers for the same area.

**Table 1:** Fibers surface area values and amount of biotin molecules per square nanometer at the fibers surface.

Overall Biotin %	Surface Area of the Fiber (m^2^/g)		Biotin Molecules/nm^2^	
	PLA	PLA/PLA-*b*-PEG	PLA	PLA/PLA-*b*-PEG
0	2.1	5.5	0	0
5	2.7	7.5	0.7	8
10	2.3	9.3	0.5	9
18	3.0	7.2	14	37

Taking all the above mentioned results into consideration, it can be concluded that the presence of PLA-*b*-PEG block copolymers enhances the migration of biotin to the fiber surface, giving rise to final fibers with larger concentrations of surface-available biotin.

### 2.3. Water Stability of Fibers

**Figure 7:**
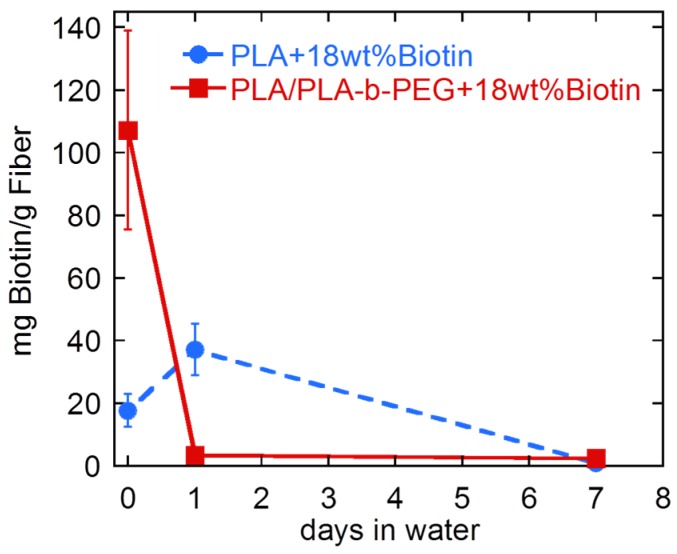
Surface available biotin of PLA and PLA/PLA-*b*-PEG fibers containing 18 wt % of biotin after being immersed in water for different periods of time.

**Figure 8:**
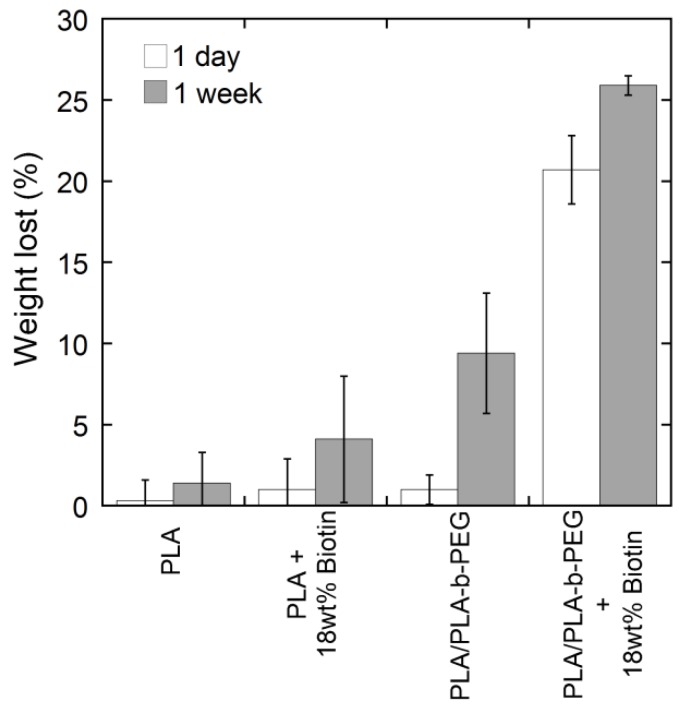
Weight loss of PLA and PLA/PLA-*b*-PEG fibers containing 0 and 18 wt % of biotin after being immersed in water for different periods of time.

Although biotin [6,16,17] or block copolymers [20,21,23] have been previously incorporated into PLA fibers, their stability in water for long periods of time has never been thoroughly investigated. To this end, fibers (PLA and PLA/PLA-*b*-PEG) containing 0 and 18 wt % of biotin were immersed in water for 24 h and one week. After removing the samples from the water and drying them, their loss of weight as well as their surface-available biotin (by competitive colorimetric assay) were measured. Results are shown in Figure 7 and Figure 8.

PLA/PLA-*b*-PEG fiber containing 18 wt % of biotin lost 97% of its surface-available biotin after being immersed in water for 24 h (Figure 7). The amount of surface-available biotin dropped from 107.2 ± 31.8 to 3.4 ± 1.0 mg biotin/g fiber. On the contrary, in the case of the PLA sample containing 18 wt % of biotin, an unexpected increase of surface-available biotin was observed after one day of water immersion. Surface-available biotin increased from 17.7 ± 5.2 to 37.2 ± 8.2 mg biotin/g fiber, indicating that, in this case, the immersion of the fiber into the water enhanced the migration of the biotin buried inside the fiber to the surface due to its high hydrophilicity. Nonetheless, after one week in water, the amount of biotin dropped to 0.8 ± 0.2 mg biotin/g fiber. Thus, even if the migration of biotin to the surface was first enhanced when PLA fibers were immersed in water, after longer periods of time, biotin migrated from the fiber surface to the aqueous phase.

The loss of weight of the fibers after being immersed in water (Figure 8) was attributed to the migration (or dissolution) of the biotin, PLA-*b*-PEG block copolymers or both to the aqueous phase. High molecular weight PLA (the main fiber forming component) was expected to be unaffected by the presence of water due to its high hydrophobicity. Overall, PLA/PLA-*b*-PEG fibers resulted in a greater weight loss than PLA fibers. The small weight differences observed for the PLA sample are attributed to experimental errors. There was no statistical difference between the weight loss after one day and one week of water immersion in PLA samples (containing 0 and 18 wt % of biotin). On the contrary, the difference was statistically significant in the case of PLA/PLA-*b*-PEG samples (containing 0 and 18 wt % of biotin). PLA/PLA-*b*-PEG samples with no biotin lost little to no weight after one day of water immersion, whereas PLA/PEG-*b*-PEG fibers with 18 wt % of biotin lost around 20% of its original weight, which was attributed mainly to the migration of biotin to the water phase (see Figure 7). After one week of water immersion, a loss of weight of 10% and 25% were observed for PLA/PLA-*b*-PEG samples with no biotin and 18 wt % of biotin respectively, indicating that PLA-*b*-PEG block copolymers also migrated to the aqueous phase with time. As smaller molecules diffuse faster than larger ones, migration of biotin to the aqueous phase occurs just after one day, whereas PLA-*b*-PEG block copolymers require lengthier periods of exposure to the aqueous system.

Li *et al*. [6] incorporated 3 wt % of biotin into PLA fibers and proved that biotin remained on the fiber surface after washing it with phosphate buffered saline (PBS) solution. The reason for the migration of the biotin observed in this work may be due to the fact that samples were immersed in water for long time durations (one day and one week). As a conclusion, the functional hydrophilic non-water-soluble fibers presented in this work show a potential application for their use as biosensors in point-of-care diagnostics, such as paper based diagnostic devices, where short contact periods of times are required between the sensing substrate material and the sample solution [30]. Reinholt *et al.* demonstrate that PLA based electrospun nanofibers could be successfully incorporated as platform in paper-based biological lateral flow assays. By a simple adsorption of the antibodies to the nanofiber, they were able to develop an enzymatic sandwich immunoassay for the detection of *E. coli* [30]. A similar approach could be used in the future to functionalize the nanofibers developed in this work, where biotinylated antibodies could be strongly bound to fiber mat due to the biotin-avidin/streptavidin complex.

## 3. Materials and Methods

### 3.1. Materials

Needle Deflect point 20 g × 2 in. was purchased from Fisher Scientific Company (Atlanta, GA, USA). PLA-*b*-PEG block copolymer was synthesized by ring opening polymerization of lactide (Sigma-Aldrich, Allentown, PA, USA) in the presence of poly(ethylene glycol) methyl ether (Number average molecular weight (M_n_): 5000 g/mol, Sigma-Aldrich), using stannous octoate (Sigma-Aldrich) as catalyst [31,32,33,34]. The final block copolymer contained 10 units of lactic acid and a molar mass dispersity value of 1.05 determined by nuclear magnetic resonance (NMR) and gel permeation chromatography (GPC) (see Supplementary Materials for detailed synthesis procedure and characterization, Figure S1). Anhydrous DMF 99.8%, PBS and Tween 20 were all purchased from Sigma-Aldrich. PLA 4043D (M_W_: 1.5 × 10^5^ g/mol, molar-mass disperisity (D): 1.81) was purchased from NatureWorks (Blair, NE, USA). Pierce™ Biotin HABA/avidin Quantitation Kit was purchased from ThermoFisher Scientific (Rockford, IL, USA).

### 3.2. Preparation of Electrospinning Solutions

Two homogeneous electrospinning solution sets were prepared by dissolving them in DMF for at least 1.5 h at 70 ± 5 °C; (1) high molecular weight PLA and biotin (0, 5, 10, and 18 wt % in the final fiber) and (2) high molecular weight PLA, PLA-*b*-PEG block copolymer and biotin (0, 5, 10 and 18 wt % in the final fiber). All initial solutions contained a total of 22 wt % of PLA. The amount PLA-*b*-PEG block copolymers added was adjusted to obtain a final fiber with a composition of 12 wt % of PEG.

### 3.3. Electrospinning

We utilized the modified electrospinning apparatus and procedure described previously by Buttaro *et al*. [21]. A high voltage supply (Gamma high Voltage Research) provided 15 kV to the needle tip and a grounded copper collector was placed 10 cm away. The solution feed rate was maintained at 10 μL/min using a programmable PHS Ultrasyringe pump (Harvard Apparatus, Holliston, MA, USA). During electrospinning, a shielded heating unit preheated to 70 ± 5 °C controlled the syringe temperature. A heat gun (Master Appliances Corp., Racine, WI, USA) was used to keep the needle temperature at 70 ± 5 °C. The temperature at the needle was verified approximately every 30 min using a digital thermometer (Fisher Scientific Company, Pittsburgh, PA, USA). The polymer solution was spun for 45 min to 1 h obtaining a fiber mat with a thickness of approximately 400 μm.

### 3.4. FESEM Imaging and Energy Dispersive Spectroscopy (EDS)

Images were taken at 1 kV voltage using a Zeiss 1550 FESEM (Zeiss, Oberkochen, Germany) to analyze fiber morphology. Samples were left uncoated during imaging. ImageJ™ open source software (National Institutes of Health, Bethesda, MD, USA) was used on the FESEM images to measure the mean average fiber diameters. Fifty measurements were taken for each sample from three separate images.

EDS was used was used to confirm the presence of biotin in the fibers and to analyze its distribution. Sulfur atom was used as an indicator of biotin molecules in the spun fiber. Polymer and biotin structures (Figure S2) as well as EDS spectra (Figures S3 and S4) are shown in Supplementary Materials. Five spectra were collected at 10 kV for 30 s in random areas (11 × 7.5 μm) of each sample and S/C atom ratio was quantified in all of them.

### 3.5. Colorimetric Assay

A competitive colorimetric assay was performed based on the work carried out by Li and co-workers [17] to quantify the amount of available biotin at the fibers surface. A Lambda 35 UV/Vis Spectrophotometer from Perkin Elmer (Beaconsfield, UK) was employed to measure the change in absorbance at 500 nm. Experiments were performed using a Pierce™ Biotin Quantitation Kit from ThermoFisher Scientific (Rockford, IL, USA). Reconstitution of the HABA/avidin solution was performed as per the manufacturer protocol. First, the absorbance of the HABA/avidin solution in PBS Buffer was measured at 500 nm. Then, a pre-weighted small piece of the fiber mat was placed into the cuvette and shaken for three minutes, the fiber was removed and the absorbance of the solution was measured again. The absorbance measurement was repeated until the obtained value was stable. The surface-available biotin was calculated using the following equation [17]:

(1) $$\text{Surface}\;\text{available}\;\text{biotin}\;(\text{mg}\;\text{biotin}/\text{g}\;\text{fiber})=\left(A_{500}^{0}-A_{500}\right)\left(\frac{{\text{Mw}}_{\text{biotin}}V}{\varepsilon \text{bW}}\right)\times 10^{3}$$

where, $A_{500}^{0}$ is the absorbance of the solution prior to the addition of nanofiber; A_500_ is the absorbance of the solution after reaction with nanofiber; Mw_biotin_ is the molecular weight of the biotin (244.3 g/mol); V is the volume of the solution (L); b is the cuvette path length (1 cm); ε is the extinction coefficient of the HABA/avidin complex at 500 nm (3.4 × 10^3^ L/(mol cm)); and W is fiber mat weight (g).

### 3.6. Water Stability

PLA and PLA/PLA-*b*-PEG fibers with 0 and 18 wt % of biotin were cut into 3 × 1 cm rectangles, weighed and immersed in individual vials with 15 mL of DI water for one day and one week. Three replicates of each sample were prepared. Once the samples were removed from the water, they were dried in a vacuum oven at room temperature for 24 h and reweighed to determine their weight loss. Another competitive colorimetric assay was performed on the fibers to assess the amount of biotin retained at the fiber surface.

## 4. Conclusions

In this work, biotin surface functionalized hydrophilic non-water soluble PLA nanofibers were produced for their potential use as biosensors. Varying concentrations of biotin (up to 18 wt %) were incorporated into PLA/DMF spinning dopes, and PLA/PLA-*b*-PEG/DMF spinning dopes. Fibers containing biotin and PLA-*b*-PEG block copolymers showed smaller fiber diameters and rougher morphologies than PLA fibers containing just biotin. In addition, the amount biotin at the fiber surface dramatically increased when PLA-*b*-PEG block copolymers were added, obtaining fibers with up to 60% of their total amount of biotin available at the surface. Finally, water stability test show that after immersing the fibers in water for long durations of time, both biotin and PLA-*b*-PEG block copolymers tended to migrate to the aqueous phase. As a conclusion, the functional hydrophilic nanofibers created in this work show potential application for use in point-of-care diagnostics where short contact times are required between the sensing substrate material and the sample solution.

## Supplementary Materials

The following are available online at www.mdpi.com/1996-1944/9/1/47/s1, Synthesis and characterization method of PLA-*b*-PEG block copolymers, Energy-dispersive X-ray spectroscopy (EDS) and fiber surface area and biotin molecules per nm^2^ calculations. Figure S1: ^1^H-NMR spectra of a PLA-*b*-PEG block copolymer; Figure S2: PLA, PLA-*b*-PEG block copolymer and biotin molecule structures; Figure S3: EDS spectra of PLA/PLA-*b*-PEG fiber containing 18 wt % of biotin; Figure S4: EDS spectra of PLA/PLA-*b*-PEG fiber containing 0 wt % of biotin.
