# Supplementary material for: Surface Functional Poly(lactic Acid) Electrospun Nanofibers for Biosensor Applications
Source: Materials (Basel). 2016 Jan 14;9(1):47. doi: 10.3390/ma9010047 (PMC5456551; doi:10.3390/ma9010047)
Supplement: Supplementary file 1 [file materials-09-00047-s001.pdf]

Supplementary Materials: Surface Functional Poly(lactic acid) Electrospun Nanofibers for Biosensor Applications

Edurne González, Larissa M. Shepherd, Laura Saunders and Margaret W. Frey

Synthesis and Characterization of Poly(lactic acid)-bock-poly(ethylene glycol) (PLA-*b*-PEG) Block Copolymers

PLA-*b*-PEG block copolymers were synthesized by ring opening polymerization of lactide in the presence of poly(ethylene glycol) methyl ether (MeOPEG, number average molecular weight (M_n): 5000 g/mol), using stannous octoate ($Sn(Oct)_2$) as catalyst [1,2]. Polymerization reaction was performed at 140 °C in a three neck round bottom flak under continuous N_2 flow. First, MeOPEG and Lactide (previously recrystalized from ethyl acetate and dried under vacuum at 40 °C [3]) were added to the round bottom flask, the temperature was increased to 90 °C and the reaction mixture was stir under N_2 atmosphere until both components were completely melted (1 h). Then, $Sn(Oct)_2$ solution in toluene (50 wt %, being the amount of $Sn(Oct)_2$ 5 wt % respect to the total MeOPEG and lacite) was added to the reaction mixture and the temperature was increased to 140 °C to start the reaction. After 16 h, polymerization reaction was stopped by cooling it to 50 °C. The reaction product was dissolved in dichloromethane and precipitated in cold diethyl ether (by adding the solution dropwise). Finally, the polymer was isolated by vacuum filtration and dried at 40 °C under vacuum overnight.

Molecular weight and molar mass dispersity of the synthesized copolymers was determined by Gel Permeation Chromatography (GPC) and Nuclear Magnetic Resonance (NMR). A Waters Ambient-Temperature GPC equipped with a Waters 410 differential refractive index detector (Millipore Corporation, Milford, MA, USA) was used. Tetrahydrofuran (THF) was used as mobile phase with a flow rate of 1 mL/min. Obtained molecular weight values were referred to polystyrene standards. ^{1}H-NMR experiments were recorded at room temperature with a INOVA 400 spectrometer (Varian Inc., Palo Alto, CA, USA) operating at 400 MHz. $CDCl_3$ was used as solvent and tetramethylsilane (TMS) as an internal reference. The Lactide/Ethylen glycol (LA/EG) ratio of the block copolymer was determined from the integration of ^{1}H-NMR resonances belonging to the PEG blocks at 3.6 ppm (-O-CH_2-CH_2- singlet) and to the PLA blocks at 5.2 ppm (-CH quartet), as previously described in the literature [2–4]. M_n of the block copolymers was calculated according to the following equations:

$$M_n = DP_{PEG} \times 44 + DP_{PLA} \times 72 \quad (S1)$$

$$DP_{PEG} = M_{nPEG}/44 \quad (S2)$$

$$DP_{PLA} = DP_{PEG} \times (LA/EG) \quad (S3)$$

where DP_{PEG} and DP_{PLA} are the polymerization degree of PEG and PLA chains respectively; and 44 and 72 are the molecular weight of EG and LA units respectively. Figure S1 shows the PLA-*b*-PEG block copolymer spectra.

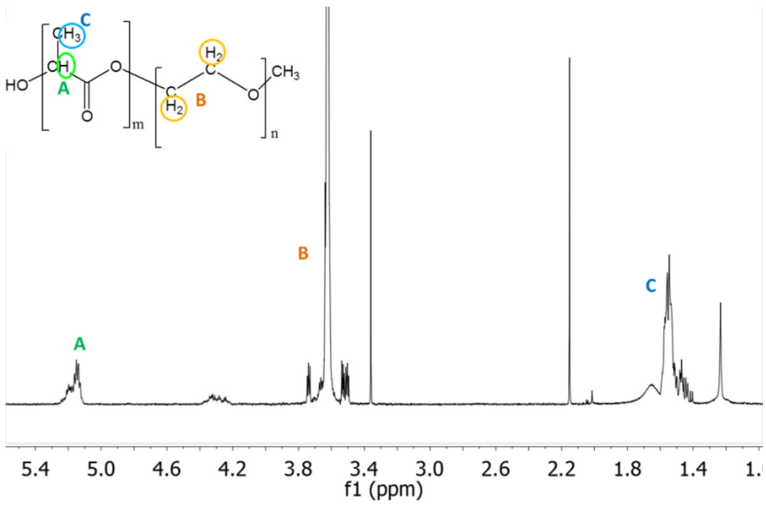

Figure S1. ^{1}H-NMR spectra of a PLA-*b*-PEG block copolymer.

Energy-Dispersive X-ray Spectroscopy (EDS)

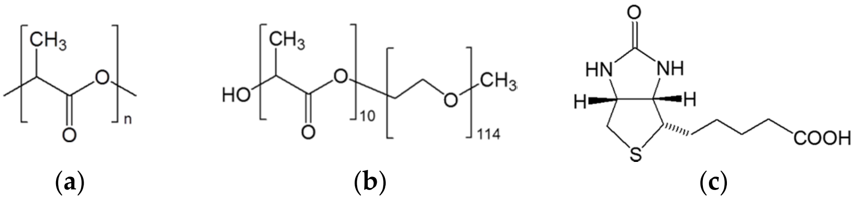

Figure S2. (**a**) PLA polymer structure; (**b**) PLA-*b*-PEG block copolymer structure and (**c**) Biotin molecule.

As sulfur atom is only presented in biotin molecule, it can be used as indicator of the presence of biotin in the sample. Figures S3 and S4 show the EDS spectra of the PLA/PLA-*b*-PEG fiber containing 0 and 18 wt % of biotin. No S signal was observed when no biotin was present in the sample.

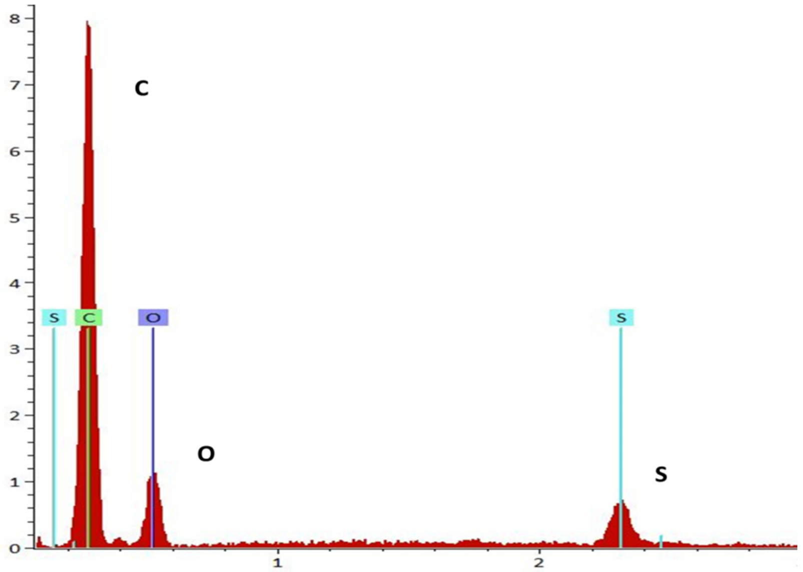

Figure S3. EDS spectra of PLA/PLA-*b*-PEG fiber containing 18 wt % of biotin.

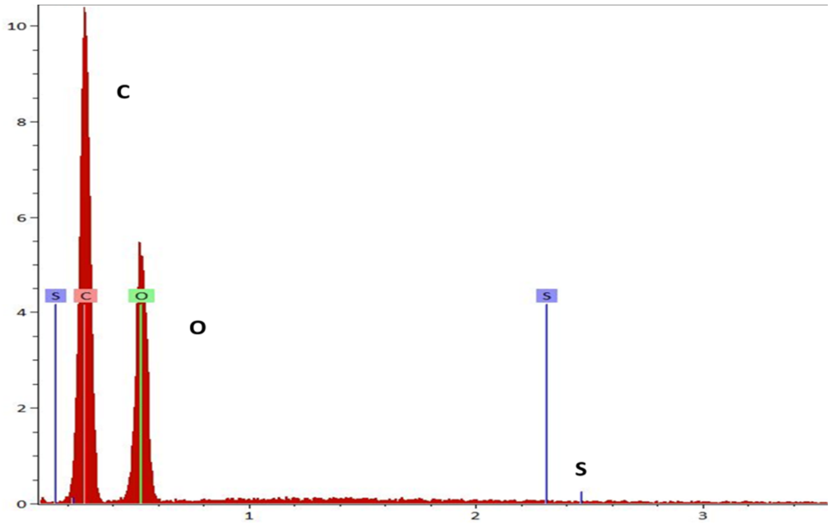

Figure S4. EDS spectra of PLA/PLA-*b*-PEG fiber containing 0 wt % of biotin.

Surface Area and Biotin Molecules per nm²

Surface area (SA) of fibers was calculated according to the following equation:

$$SA\ (m^2/g) = \frac{A_f(cm^2)}{m_f(g)} \times 10^{-4} \quad (S4)$$

where A_f is the area of the fiber (cm^2); and m_f is the fiber mass (g).

A_f was calculated according to Equation (S5):

$$A_f(cm^2) = 2\pi rh \quad (S5)$$

where r is the fiber radius (cm); and h is the fiber length (cm). Fiber diameter (and therefore radius) was measured using the SEM images as explained in Section 3.4 of the experimental work. Fiber length (h) was calculated using Equation (S8):

$$m_f = \rho \times v \quad (S6)$$

$$v = \pi r^2 h \quad (S7)$$

$$h = \frac{m_f/\rho}{\pi r^2} \quad (S8)$$

where ρ is the fiber density (g/cm^3); and v is the volume of the fiber (cm^3). PLA density value (1.2 g/cm^3) was used for calculations.

Therefore, measuring the fiber mat sample mas (m_f) and fiber radius (r), surface area of the fiber mat (SA, area of the fiber mat per gram of the sample) can be calculated using Equations (S4), (S5) and (S8).

Biotin molecules per nm^2 were calculated using Equation (S9):

$$\frac{\text{Biotin molecules}}{nm^2} = \frac{g_{sb}/g_f \times N_A}{M_{Wb} \times SA \times 10^{18}} \quad (S9)$$

where g_{sb}/g_f is the grams of surface available biotin per fiber gram calculated by colorimetric assay; M_{Wb} is the molar mass of biotin (244.3 g/mol); SA is the surface area (m^2/g) calculated from section 3.5 of experimental work; and N_A is the Avogadro´s constant (6.022 × 10^{23} molecule/mol); 10^{18} is the conversion number to change the units of the fiber area from m^2 to nm^2.

References

1. Riley, T.; Stolnik, S.; Heald, C.R.; Xiong, C.D.; Garnett, M.C.; Illum, L.; Davis, S.S.; Purkiss, S.C.; Barlow, R.J.; Gellert, P.R. Physicochemical evaluation of nanoparticles assembled from poly(lactic acid)-poly(ethylene glycol) (PLA-PEG) block copolymers as drug delivery vehicles. *Langmuir* **2001**, *17*, 3168–3174.
2. Li, S.; Vert, M. Synthesis, characterization, and stereocomplex-induced gelation of block copolymers prepared by ring-opening polymerization of L(D)-lactide in the presence of poly(ethylene glycol). *Macromolecules* **2003**, *36*, 8008–8014.
3. Venkatraman, S.S.; Jie, P.; Min, F.; Freddy, B.Y.C.; Leong-Huat, G. Micelle-like nanoparticles of PLA-PEG-PLA triblock copolymer as chemotherapeutic carrier. *Int. J. Pharm.* **2005**, *298*, 219–232.
4. Molina, I.; Li, S.; Martinez, M.B.; Vert, M. Protein release from physically crosslinked hydrogels of the PLA/PEO/PLA triblock copolymer-type. *Biomaterials* **2001**, *22*, 363–369.
